# Acute reversible inactivation of the bed nucleus of stria terminalis induces antidepressant-like effect in the rat forced swimming test

**DOI:** 10.1186/1744-9081-6-30

**Published:** 2010-06-01

**Authors:** Carlos C Crestani, Fernando HF Alves, Fernando MA Correa, Francisco S Guimarães, Sâmia RL Joca

**Affiliations:** 1 Department of Pharmacology, School of Medicine of Ribeirão Preto, University of São Paulo, 14049-900, Ribeirão Preto, SP, Brazil; 2 Laboratory of Pharmacology, Department of Physics and Chemistry, School of Pharmaceutical Sciences of Ribeirão Preto; University of São Paulo, 14040-903, Ribeirão Preto, SP, Brazil

## Abstract

**Background:**

The bed nucleus of stria terminalis (BNST) is a limbic forebrain structure involved in hypothalamo-pituitary-adrenal axis regulation and stress adaptation. Inappropriate adaptation to stress is thought to compromise the organism's coping mechanisms, which have been implicated in the neurobiology of depression. However, the studies aimed at investigating BNST involvement in depression pathophysiology have yielded contradictory results. Therefore, the objective of the present study was to investigate the effects of temporary acute inactivation of synaptic transmission in the BNST by local microinjection of cobalt chloride (CoCl_2_) in rats subjected to the forced swimming test (FST).

**Methods:**

Rats implanted with cannulae aimed at the BNST were submitted to 15 min of forced swimming (pretest). Twenty-four hours later immobility time was registered in a new 5 min forced swimming session (test). Independent groups of rats received bilateral microinjections of CoCl_2 _(1 mM/100 nL) before or immediately after pretest or before the test session. Additional groups received the same treatment and were submitted to the open field test to control for unspecific effects on locomotor behavior.

**Results:**

CoCl_2 _injection into the BNST before either the pretest or test sessions reduced immobility in the FST, suggesting an antidepressant-like effect. No significant effect of CoCl_2 _was observed when it was injected into the BNST immediately after pretest. In addition, no effect of BNST inactivation was observed in the open field test.

**Conclusion:**

These results suggest that acute reversible inactivation of synaptic transmission in the BNST facilitates adaptation to stress and induces antidepressant-like effects.

## Background

The bed nucleus of stria terminalis (BNST) is a limbic forebrain structure situated ventrally to the lateral septal nucleus and dorsally to the preoptic area of the hypothalamus [1,2]. It has extensive reciprocal connections with other limbic structures as well as with brainstem autonomic nuclei [2-5], and it is an import relay station for the integration of information from brain regions associated with the control of emotional, cognitive, autonomic, endocrine and behavioral responses [2,6-13].

Several studies have suggested that the BNST mediates behavioral responses to acute and chronic aversive stimuli [5,14]. This is supported by reports that the BNST is activated in response to stress [15-18] and modulates anxiety-related behaviors in several animal models [5,10,19,20]. Moreover, the BNST could also mediate behavioral adaptation to chronic stress exposure [21-24]. Inappropriate adaptation to stress is thought to compromise the organism's coping mechanisms, which have been implicated in the etiology of stress-related disorders, such as posttraumatic stress disorder (PTSD) and depression [25-27].

BNST involvement in the activation and termination of the hypothalamo-pituitary-adrenal (HPA) axis response to stress has been well documented in the literature [6,28-30]. Activation of the HPA axis is a primary mechanism for maintaining homeostasis in response to stress. Although adaptative in nature, glucocorticoids secretion is tightly regulated since prolonged exposure to their effects can lead to serious metabolic, immune, and psychological dysfunction. Dysfunction in forebrain limbic regions that exert control over the HPA axis, such as the amygdala, hippocampus and medial prefrontal cortex (MPFC) [31,32], has been implicated in the etiology of stress-related disorders, including PTSD and depression, which often exhibit HPA axis abnormalities [33,34]. Substantial information from these forebrain regions are integrated in the BNST that could either excite or inhibit HPA activity depending on the region of the BNST targeted [6].

Failure of coping mechanisms has been recognized as a major factor precipitating depressive episodes in humans [27,35,36]. BNST role in stress adaptation and its connections with other limbic structures traditionally related to depression, such as the hippocampus and the MPFC, has made it a subject of study in different behavioral paradigms aimed at investigating the neurobiology of depression. In fact, the BNST is activated by stressful stimuli that induce depressive-like behavior in rodents [16,37,38] and this can be attenuated by systemic antidepressant-treatment [37], corroborating the idea that BNST dysfunction could contribute to the pathophysiology of depression. However, the studies aimed at investigating this hypothesis through local inactivation of BNST have yielded contradictory results. For example, while chemical lesions of the BNST induced antidepressant-like effects in the rat learned helplessness model [16,19], electrolytic lesions of the BNST increased depressive-like behavior in the rat forced swimming test (FST) [39-41]. The reasons for these contradictory results are not clear, but could involve the different animal models used or methodological differences in the lesions employed (size, time of recovery or nature of the lesion - chemical versus electrical). In addition, irreversible lesions can also destroy fibers of passage and induce local plastic changes [42]. Another disadvantage of lesion techniques is that they do not allow the identification of the precise moment when the disruption of BNST activity affects the development of the depressive-like behavior (during the pretest or during the test). Considering that BNST is interconnected with brain structures implicated in learning and memory of aversive events, such as the MPFC, the hippocampus and the amygdale [43], it would be interesting to investigate the participation of such cognitive mechanisms in the development of stress-induced behavioral consequences in the FST mediated by BNST.

Therefore, considering the contradictory results regarding the role of the BNST in the modulation of depressive-like behavior, and the fact that the time point of BNST influence in the FST has never been evaluated, the objective of the present study was to investigate the effects of temporary acute inactivation of synaptic transmission in the BNST, at different time points (before pretest, after pretest or before test), by local microinjection of cobalt chloride (CoCl_2_) in rats submitted the FST. This drug reduces calcium pre-synaptic influx [44] and causes a reversible inhibition of neurotransmitter release with a consequent synaptic blockage, without affecting passage fibers.

## Methods

### Animals

Male Wistar rats weighing 230-250 g at the beginning of each experiment were housed in pairs in a temperature-controlled room (24 ± 1°C) under standard laboratory conditions with free access to food and water and a 12 h light/12 h dark cycle (lights on at 06:30 h a.m.). Procedures were conduct in conformity with the Brazilian Society of Neuroscience and Behavior guidelines for the care and use of laboratory animals, which are in compliance with international laws and politics. The protocols described herein have been approved by the local Ethical Committee and all efforts were made to minimize animal suffering.

### Drugs

The following drugs were used: cobalt chloride (CoCl_2_; Sigma, St Louis, Missouri, USA), tribromoethanol (Aldrich, St Louis, Missouri, USA) and urethane (Sigma, St Louis, Missouri, USA). CoCl_2 _was dissolved in sterile artificial cerebrospinal fluid (ACSF: 100 mM NaCl; 2 mM Na3PO4; 2.5 mM KCl; 1 mM MgCl2; 27 mM NaHCO3; 2.5 mM CaCl2; pH = 7.4). Tribromoethanol and urethane were dissolved in saline 0.9%.

### Stereotaxic surgery and intracerebral drug administration

Seven days before the experiment, animals were anaesthetized with tribromoethanol (250 mg/kg, i.p.) and fixed in a stereotaxic frame. After scalp anesthesia with 2% lidocaine, the skull was surgically exposed and stainless steel guide cannulae (26 G) were implanted bilaterally in the BNST using a stereotaxic apparatus (Stoelting, Wood Dale, Illinois, USA). Coordinates for cannula implantation (AP = +8.6 mm from interaural coordinate; L = +4 mm from the medial suture, V = -5.8 mm from the skull with a lateral inclination of 23°) were selected from the rat brain atlas of Paxinos and Watson [44]. The cannulae tips were 1 mm above the site of injection and the cannulae were attached to the skull bone with stainless steel screws and acrylic cement. An obturator inside the guide cannulae prevented obstruction. After surgery, the animals received a poly-antibiotic (Pentabiotico^®^, Fort Dodge, Brazil), with streptomycins and penicillins, to prevent infection and a nonsteroidal anti-inflammatory, flunixine meglumine (Banamine^®^, Schering Plough, Brazil), for post- operation analgesia.

The needles (33G, Small Parts, Miami Lakes, FL, USA) used for microinjection into the BNST were 1 mm longer than the guide cannulae and were connected to a 2 μL syringe (7002-H, Hamilton Co., Reno, NV, USA) through PE-10 tubing. A volume of 100 nL/side was injected in 1 minute using an infusion pump (Kd Scientific, Holliston, MA, USA). The movement of an air bubble inside the polyethylene catheter confirmed drug flow.

### Forced swimming test (FST)

The procedures for the FST, a widely used behavioral test for the detection of antidepressant-like effects, were similar to those described earlier [45-48]. Animals were initially placed individually to swim in plastic cylinders (30 cm of diameter by 40 cm in height containing 25 cm of water at 24 ± 1°C [49] for 15 min (pretest). They were then removed and allowed to dry in a separate cage before returning to their home cages. Twenty-four hours later the animals were submitted to a 5 min session of forced swimming session (test). During this session the total amount of time in which animals remained immobile (except for small limb movements necessary for floating) were recorded by an observer that was blind to the treatments. The water was changed after each trial to avoid the influence of alarm substances.

### Open field test

Independent groups of animals were submitted to the open field test in order to investigate if the treatments used induced any significant motor effect, which would interfere in the FST results. The animals were placed individually in the center of an open circular arena (72 cm in diameter with a 50 cm high Plexiglas wall) located in a sound-attenuated, temperature-controlled room, illuminated with three 40W fluorescent bulbs. The animals were left in the arena for 10 minutes. Their exploratory activity was videotaped and the behavioral analysis was blindly performed with the help of the Ethovision software (version 1.9; Noldus, the Netherlands). This software detects the position of the animal in the open arena and calculates the distance moved.

### Histological analysis

After the behavioral tests, animals were anesthetized with urethane (1.25 g/kg, i.p.) and then 100 nl of 1% Evan's blue dye was injected into the BST as a marker of injection site. Following that, they were perfused through the left ventricle of the heart with isotonic saline followed by 10% formalin solution. The brains were removed and after a minimum period of 3 days immersed in a 10% formalin solution, 40 μm sections were obtained in a Cryostat (Cryocut 1800). The injection sites were identified on diagrams from the Paxinos and Watson's atlas [50]. Rats that had received injections outside the aimed area were excluded from analysis.

### Experimental design

#### Experiment 1: effects of CoCl_2 _injection into the BNST of rats submitted to the FST

Animals were randomly assigned to three independent groups which received bilateral injection into the BNST of either 100 nL of vehicle (ACSF) [51] or 1 mM/100 nL of CoCl_2 _[10,51] and were submitted to FST. The first group of animals received the microinjections into the BNST 10 minutes before the pretest session (ACSF: n = 5 and CoCl_2_: n = 6). The second group received the microinjections into the BNST immediately after the end of pretest session (ACSF: n = 6 and CoCl_2_: n = 6). Finally, the third group received the microinjections into the BNST 10 minutes before the test session (ACSF: n = 6 and CoCl_2_: n = 7). Additional groups received the microinjections into structures surrounding the BNST before the pretest (ACSF: n = 4 and CoCl_2_: n = 3) or before the test (ACSF: n = 3 and CoCl_2_: n = 5).

#### Experiment 2: effects of CoCl_2 _injection into the BNST of rats submitted to the open field test

Animals received the microinjections of either 100 nL of vehicle (ACSF, n = 6) or 1 mM/100 nL of CoCl_2 _(n = 6) [10,12] into the BNST and were submitted to the open field test 10 min later.

### Statistical analysis

The results of the FST and from open-field test were analyzed using unpaired *Student*-t test. Probability less than 0.05 was accepted as significant.

## Results

### Determination of microinjection sites

**Figure 1:**
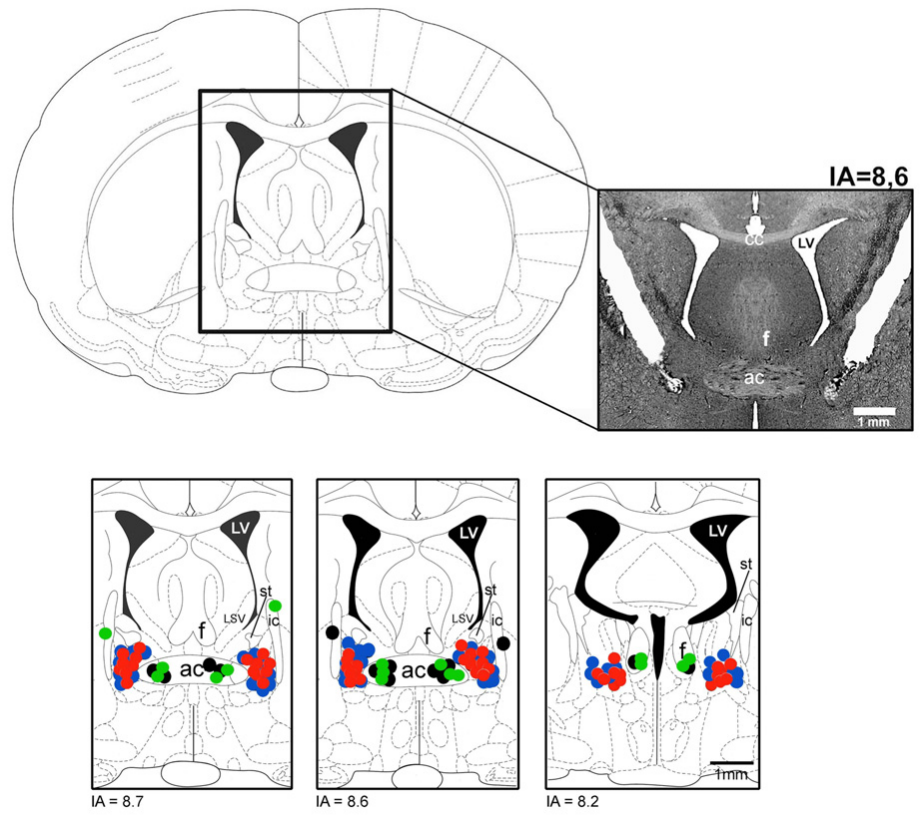
**Photomicrograph of a coronal brain section from a representative rat showing bilateral injection sites in the BNST and a diagrammatic representation based on the rat brain atlas of Paxinos and Watson **[50]**, indicating injection sites of ACSF (blue circles) and CoCl_2 _(red circles) into the BNST and ACSF (black circles) and CoCl_2 _(green circles) into structures surrounding the BNST**. IA - Interaural coordinate, ac - anterior commissure, cc - corpus callosum, ic - internal capsule, LSV - lateral septal ventral, LV - lateral ventricle, st - stria terminalis and f - fornix.

A representative photomicrograph of a coronal brain section depicting bilateral microinjection sites in the BST of one representative animal is presented in Figure 1. Moreover, diagrammatic representation showing microinjection sites of ACSF and CoCl_2 _into the BNST and in structures surrounding the BNST are also shown in Figure 1.

### Experiment one: effects of CoCl_2 _injection into the BNST of rats submitted to the FST

**Figure 2:**
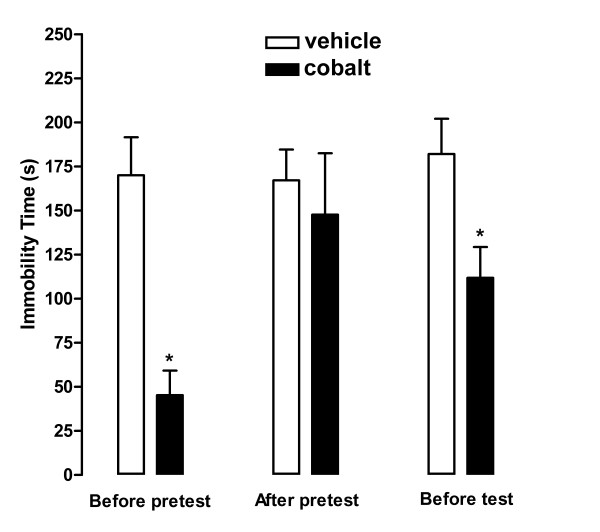
**Total immobility time during the 5 min session of the forced swimming test (test) shown by animals treated with vehicle (ACSF, open columns) or CoCl_2 _(cobalt, filled columns) into the BNST**. Intra-BNST injection of CoCl_2 _(1 mM/100 nL) before pretest or before test reduced immobility time in the rat forced swimming test. Data are expressed as mean ± S.E.M. (n = 5-7/group). * indicates significant difference from respective vehicle-treated group (*P *< 0.05, *Student*-t test).

Injection of CoCl_2 _into the BNST before the pretest (170 ± 21 vs 46 ± 13 s, t_9 _= 5.024, *P *< 0.001) or the test (182 ± 20 vs 111 ± 17 s, t_10 _= 2.627, *P *< 0.05) sessions induced a significant reduction of immobility time in the FST (Figure 2). There was no significant statistical difference between vehicle and CoCl_2_-treated groups that received the injection into the BNST immediately after the pretest (167 ± 17 vs 148 ± 33 s, t_10 _= 2.627, *P *< 0.05) (Figure 2). Immobility time obtained in animals that received CoCl_2 _before the pretest or before the test was significantly different (t_11 _= 2.9, P < 0.05). Injection of CoCl_2 _into structures surrounding the BNST, such as anterior commissure, internal capsule or fornix, before the pretest (173 ± 11 vs 182 ± 23 s, t_5 _= 0.385, *P *> 0.05) or the test (189 ± 19 vs 162 ± 12 s, t_6 _= 1.244, *P *> 0.05) did not affect immobility time in the FST.

### Experiment two: effects of CoCl_2 _injection into the BNST of rats submitted to the open field test

**Figure 3:**
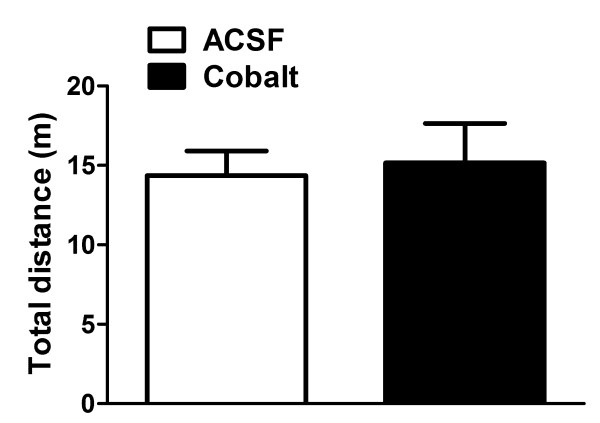
**Total distance travelled by rats exposed to the open field test shown by animals treated with vehicle (ACSF, white bar, n = 6) or CoCl_2 _(cobalt, black bar, n = 6) into the BNST**. Intra-BNST injection of CoCl_2 _(1 mM/100 nL) before test did not modify the rat locomotor activity in the open field test (*P *> 0.05, unpaired *Student*-t test).

Analysis of total distance travelled in the open-field test did not show a significant effect of BNST treatment with CoCl_2 _(14 ± 2 vs 15 ± 3 m, t_10 _= 0.27, *P *> 0.05), when compared with animals treated with vehicle (Figure 3).

## Discussion

The FST is probably the most frequently used animal model predictive of antidepressant activity [45]. It is based on the observation that rodents exposed to an enclosed cylinder filled with water perform escape oriented behaviors for few minutes and, afterwards, assume a posture of immobility which is of shorter duration in animals that had received antidepressant treatment [45,47,48]. In the present study, the results showed that intra-BNST injection of CoCl_2 _decreased the immobility time in this model when microinjected before pretest or before test, whereas treatment after pretest did not have any effect. These effects do not rely on unspecific motor changes, since CoCl_2 _administration into the BNST did not modify animal's locomotor activity in the open field test. Therefore, these results are indicative of an antidepressant-like effect induced by transient blockage of synaptic transmission in the BNST during the pretest or test sessions in the rats submitted to the FST.

Contradictory results regarding BNST role in animal models of depression have been previously described [39-41]. It has been reported, for example, that chronic electrolytic lesion of the BNST increases rather than decreases the behavioral consequences produced by the FST [38-40]. However, chronic electrolytic lesion destroy not only intrinsic BNST neurons, but also fibers of passage that project through it on their way to other structures. On the other hand, chemical lesions of the BNST, which spares fibers of passage, induced antidepressant-like effects in the rat learned helplessness model of depression [19]. The present study, by inducing a reversible inactivation of synaptic transmission that spares fibers of passage, corroborates the previous results in the learned helplessness model and suggests that this experimental approach (CoCl_2_-induced inactivation) could be more useful to unveil the specific role of BNST neurons in FST model.

It should be noticed, however, that inconsistent findings regarding BNST participation in the modulation of depressive-like behaviors could be related to the fact that BNST is a cluster of 12 nuclei, which can be divided into anterior and posterior subdivisions, each containing several nuclei, which differ in their projection pattern and neurochemical identity [52]. In this regard, depending on the extension of the lesion or the drug distribution into BNST, different portions of it could have been affected in different studies, thus allowing the occurrence of contradictory behavioral effects in the FST.

The BNST has long been recognized as an important structure that integrates and mediates emotional, cognitive, autonomic, endocrine and behavioral responses to stress [2,6,9,10,14,20,51,53]. Lesions as well as pre-test infusions into the BNST of compounds that disrupt its function reduce behavioral and autonomic responses to stress [10,16,51,54,55]. This is especially evident in paradigms in which behavior is influenced by long-duration stimuli and in paradigms that assess the persistent behavioral effects of even a brief stressor, but that are severe and unpredictable, such inescapable shocks [5]. Corroborating this proposal, the BNST is activated in response to several aversive stimuli [15-18] including inescapable shock exposure [16]. BNST stimulation, on the other hand, produces behavioral consequences similar to those induced by forced restraint [14] whereas its chemical lesion prevents the development of behavioral deficits that characterize the "learned helplessness" phenomenon [19]. These deficits are thought to arise from increased fear and anxiety produced by the previous exposure to inescapable shocks [56,57]. Finally, blockade of BNST noradrenergic transmission attenuates immobilization stress-induced anxiogenic-like effects [54,55,58].

Taken together, these pieces of evidence indicate that activation of BNST during stress could contribute to the development of stress-induced behavioral consequences, thus impairing adaptation in a subsequent stressful situation. In this way, BNST activation during stress pre-exposure could facilitate a hyperanxiety state that would impair adaptation to a subsequent stress exposure. This is supported, for example, by the observation that the positive effects of BNST lesions in the learned helplessness model are due to a reduction in the anxiogenic effects of pre-exposure to the inescapable shocks [19]. Moreover, behavioral manipulation that prevents learned helplessness development also reduces the anxiogenic effect and the increased BNST Fos expression caused by the previous exposure to inescapable shocks [16]. Finally, stress-induced hyperanxiety have been shown to occur in association to structural and functional changes in BNST [21-24], thus supporting the involvement of this nucleus in mechanisms of stress-induced emotional consequences.

Failure to coping with stress is an important precipitating factor in depressive illnesses [27,35,36] and antidepressants promote behavioral adaptation to stress [59]. In this context, blockade of synaptic transmission within the BNST before pre-test could reduce the stress-induced behavioral outcomes (e.g. hyperanxiety) and, thus, facilitate adaptation to the subsequent stress section, inducing antidepressant-like effects [19]. Moreover, considering that BNST is uniquely positioned to receive emotional and learning associated informations and to integrate these into the reward/motivation circuitry [43], its inactivation before the test might have induced antidepressant-like effect by increasing motivation and goal-directed behavior to aimed at performing escape from the swimming stress.

It could also be speculated that the reduced immobility observed during the test could have been a consequence of learning and memory impairments induced by BSNT inactivation during pre-test and test, respectively. However, previous results from the literature showed that BNST lesions did not impair navigational learning and memory in the Morris water Maze [40], thus questioning the aforementioned suggestion. Despite that, the antidepressant-like effect reported after BNST blockage in the present study cannot be completely dissociated from effects in the cognitive performance. In fact, cognitive mechanisms have been implicated in the neurobiology of depression and antidepressant response, since they might interfere with stress adaptation and biases in the processing of negative affect [60].

Finally, it can be speculated that the deleterious effects of stress could also be mediated, at least in part, by dysregulation (e.g., overactivation) of the HPA axis [61]. Activation of the HPA axis is a primary mechanism for maintaining homeostasis in response to stress. Neurons in the paraventricular nucleus of the hypothalamus (PVN) synthesize corticotropin-releasing hormone (CRH), which is released into the hypophysial portal system and trigger adrenocorticotropin (ACTH) secretion from the anterior pituitary. ACTH stimulates the secretion of glucocorticoids from the adrenals into the circulation to mobilize energy stores, maintain blood pressure, and exert negative feedback at the HPA brain and pituitary sites (for review, see [9]). Glucocorticoids secretion needs to be tightly regulated since prolonged exposure to their effects can lead to serious metabolic, immune, and psychological dysfunction. The BNST has a central role in controlling HPA axis activity [6,28,30], which can be dysfunctional in depression [31-34]. Considering that glucocorticoids facilitate whereas adrenalectomy impairs the expression of the depressive-like behavior in the FST [62-64], it is also possible that BSNT inactivation might have attenuated HPA axis activation in response to stress, and the consequent reduction in the glucocorticoid levels could have contributed to the reduced expression of the depressive-like behavior in the FST. This hypothesis, however, warrants further investigation.

## Limitations

Considering that distinct neurobiological mechanisms can be involved in the different experimental procedures used to study the neurobiology of depression, the hypothesis discussed herein should be further tested in other animal models of depression with higher face validity than the FST.

## Conclusion

In conclusion, stress-induced BNST activation could promote a bias in the processing of threatening cues which could render the animal more susceptible to the development of behavioral consequences of stress. On the other hand, BNST inactivation before stress could protect animals against emotional changes caused by previous stressful stimuli presentation, perhaps by facilitating mechanisms involved in the ability to cope with a new stressful situation. Further studies are necessary to characterize the neurotransmitters involved in these effects.

## List of abbreviations used

ACSF: artificial cerebrospinal fluid; ACTH: adrenocorticotropin hormone; BNST: bed nucleus of the stria terminalis; CoCl_2_: cobalt chloride; CRH: corticotropin-releasing hormone; FST: forced swimming test; HPA: hypothalamo-pituitary-adrenal; MPFC: medial prefrontal córtex; PVN: paraventricular nucleus of the hypothalamus; PTSD: posttraumatic stress disorder.

## Competing interests

The authors declare that they have no competing interests.

## Authors' contributions

C.C.C. and S.R.L.J. contributed to the conception and design of the study. Moreover, S.R.L.J. was responsible by analysis and interpretation of data, drafted the manuscript and continuously supervised the study. C.C.C. and F.H.F.A. were responsible for data collection and helped to draft the manuscript. F.S.G. and F.M.A.C. helped to draft the manuscript and continuously supervised the study. All authors read and approved the final manuscript.

## References

[B1] Dong HW Petrovich GD Watts AG Swanson LW Basic organization of projections from the oval and fusiform nuclei of the bed nuclei of the stria terminalis in adult rat brain J Comp Neurol 2001 436 4 430 455 10.1002/cne.1079 11447588

[B2] Forray MI Gysling K Role of noradrenergic projections to the bed nucleus of the stria terminalis in the regulation of the hypothalamic-pituitary-adrenal axis Brain Res Brain Res Rev 2004 47 1-3 145 160 10.1016/j.brainresrev.2004.07.011 15572169

[B3] Martin LJ Powers RE Dellovade TL Price DL The bed nucleus-amygdala continuum in human and monkey J Comp Neurol 1991 309 4 445 485 10.1002/cne.903090404 1918444

[B4] Shin JW Geerling JC Loewy AD Inputs to the ventrolateral bed nucleus of the stria terminalis J Comp Neurol 2008 511 5 628 657 10.1002/cne.21870 18853414 PMC2748802

[B5] Walker DL Toufexis DJ Davis M Role of the bed nucleus of the stria terminalis versus the amygdala in fear, stress, and anxiety Eur J Pharmacol 2003 463 1-3 199 216 10.1016/S0014-2999(03)01282-2 12600711

[B6] Choi DC Furay AR Evanson NK Ostrander MM Ulrich-Lai YM Herman JP Bed nucleus of the stria terminalis subregions differentially regulate hypothalamic-pituitary-adrenal axis activity: implications for the integration of limbic inputs J Neurosci 2007 27 8 2025 2034 10.1523/JNEUROSCI.4301-06.2007 17314298 PMC6673539

[B7] Crestani CC Alves FH Resstel LB Correa FM Cardiovascular effects of noradrenaline microinjection in the bed nucleus of the stria terminalis of the rat brain J Neurosci Res 2007 85 7 1592 1599 10.1002/jnr.21250 17330275

[B8] Crestani CC Alves FH Resstel LB Correa FM Both alpha1 and alpha2-adrenoceptors mediate the cardiovascular responses to noradrenaline microinjected into the bed nucleus of the stria terminal of rats Br J Pharmacol 2008 153 3 583 590 10.1038/sj.bjp.0707591 18037912 PMC2241784

[B9] Herman JP Ostrander MM Mueller NK Figueiredo H Limbic system mechanisms of stress regulation: hypothalamo-pituitary-adrenocortical axis Prog Neuropsychopharmacol Biol Psychiatry 2005 29 8 1201 1213 10.1016/j.pnpbp.2005.08.006 16271821

[B10] Resstel LB Alves FH Reis DG Crestani CC Correa FM Guimaraes FS Anxiolytic-like effects induced by acute reversible inactivation of the bed nucleus of stria terminalis Neuroscience 2008 154 3 869 876 10.1016/j.neuroscience.2008.04.007 18479825

[B11] Alves FH Crestani CC Resstel LB Correa FM Cardiovascular effects of carbachol microinjected into the bed nucleus of the stria terminalis of the rat brain Brain Res 2007 1143 161 168 10.1016/j.brainres.2007.01.057 17306779

[B12] Crestani CC Busnardo C Tavares RF Alves FH Correa FM Involvement of hypothalamic paraventricular nucleus non-N-methyl-d-aspartate receptors in the pressor response to noradrenaline microinjected into the bed nucleus of the stria terminalis of unanesthetized rats Eur J Neurosci 2009 29 11 2166 2176 10.1111/j.1460-9568.2009.06762.x 19490019

[B13] Crestani CC Alves FH Resstel LB Correa FM Bed nucleus of the stria terminalis alpha(1)-adrenoceptor modulates baroreflex cardiac component in unanesthetized rats Brain Res 2008 1245 108 115 10.1016/j.brainres.2008.09.082 18950605

[B14] Casada JH Dafny N Restraint and stimulation of bed nucleus of the stria terminalis produce similar stress-like behaviors Brain Res Bull 1991 27 2 207 212 10.1016/0361-9230(91)90069-V 1742609

[B15] Beijamini V Guimaraes FS c-Fos expression increase in NADPH-diaphorase positive neurons after exposure to a live cat Behav Brain Res 2006 170 1 52 61 10.1016/j.bbr.2006.01.025 16546272

[B16] Greenwood BN Foley TE Burhans D Maier SF Fleshner M The consequences of uncontrollable stress are sensitive to duration of prior wheel running Brain Res 2005 1033 2 164 178 10.1016/j.brainres.2004.11.037 15694921

[B17] Ma S Morilak DA Induction of FOS expression by acute immobilization stress is reduced in locus coeruleus and medial amygdala of Wistar-Kyoto rats compared to Sprague-Dawley rats Neuroscience 2004 124 4 963 972 10.1016/j.neuroscience.2003.12.028 15026136

[B18] Valles A Marti O Armario A Long-term effects of a single exposure to immobilization: a c-fos mRNA study of the response to the homotypic stressor in the rat brain J Neurobiol 2006 66 6 591 602 10.1002/neu.20252 16555238

[B19] Hammack SE Richey KJ Watkins LR Maier SF Chemical lesion of the bed nucleus of the stria terminalis blocks the behavioral consequences of uncontrollable stress Behav Neurosci 2004 118 2 443 448 10.1037/0735-7044.118.2.443 15113272

[B20] Treit D Aujla H Menard J Does the bed nucleus of the stria terminalis mediate fear behaviors? Behav Neurosci 1998 112 2 379 386 10.1037/0735-7044.112.2.379 9588484

[B21] Blundell J Adamec R The NMDA receptor antagonist CPP blocks the effects of predator stress on pCREB in brain regions involved in fearful and anxious behavior Brain Res 2007 1136 1 59 76 10.1016/j.brainres.2006.09.078 17239834

[B22] Hammack SE Cheung J Rhodes KM Schutz KC Falls WA Braas KM May V Chronic stress increases pituitary adenylate cyclase-activating peptide (PACAP) and brain-derived neurotrophic factor (BDNF) mRNA expression in the bed nucleus of the stria terminalis (BNST): roles for PACAP in anxiety-like behavior Psychoneuroendocrinology 2009 34 6 833 843 10.1016/j.psyneuen.2008.12.013 19181454 PMC2705919

[B23] Pego JM Morgado P Pinto LG Cerqueira JJ Almeida OF Sousa N Dissociation of the morphological correlates of stress-induced anxiety and fear Eur J Neurosci 2008 27 6 1503 1516 10.1111/j.1460-9568.2008.06112.x 18336570

[B24] Vyas A Bernal S Chattarji S Effects of chronic stress on dendritic arborization in the central and extended amygdala Brain Res 2003 965 1-2 290 294 10.1016/S0006-8993(02)04162-8 12591150

[B25] Nemeroff CB Bremner JD Foa EB Mayberg HS North CS Stein MB Posttraumatic stress disorder: a state-of-the-science review J Psychiatr Res 2006 40 1 1 21 10.1016/j.jpsychires.2005.07.005 16242154

[B26] Olff M Langeland W Gersons BP The psychobiology of PTSD: coping with trauma Psychoneuroendocrinology 2005 30 10 974 982 10.1016/j.psyneuen.2005.04.009 15964146

[B27] Southwick SM Vythilingam M Charney DS The psychobiology of depression and resilience to stress: implications for prevention and treatment Annu Rev Clin Psychol 2005 1 255 291 10.1146/annurev.clinpsy.1.102803.143948 17716089

[B28] Choi DC Furay AR Evanson NK Ulrich-Lai YM Nguyen MM Ostrander MM Herman JP The role of the posterior medial bed nucleus of the stria terminalis in modulating hypothalamic-pituitary-adrenocortical axis responsiveness to acute and chronic stress Psychoneuroendocrinology 2008 33 5 659 669 10.1016/j.psyneuen.2008.02.006 18378095 PMC3641575

[B29] Jankord R Herman JP Limbic regulation of hypothalamo-pituitary-adrenocortical function during acute and chronic stress Ann N Y Acad Sci 2008 1148 64 73 10.1196/annals.1410.012 19120092 PMC2637449

[B30] Morilak DA Barrera G Echevarria DJ Garcia AS Hernandez A Ma S Petre CO Role of brain norepinephrine in the behavioral response to stress Prog Neuropsychopharmacol Biol Psychiatry 2005 29 8 1214 1224 10.1016/j.pnpbp.2005.08.007 16226365

[B31] Koenigs M Grafman J The functional neuroanatomy of depression: distinct roles for ventromedial and dorsolateral prefrontal cortex Behav Brain Res 2009 201 2 239 243 10.1016/j.bbr.2009.03.004 19428640 PMC2680780

[B32] Pittenger C Duman RS Stress, depression, and neuroplasticity: a convergence of mechanisms Neuropsychopharmacology 2008 33 1 88 109 10.1038/sj.npp.1301574 17851537

[B33] de Kloet CS Vermetten E Geuze E Kavelaars A Heijnen CJ Westenberg HG Assessment of HPA-axis function in posttraumatic stress disorder: pharmacological and non-pharmacological challenge tests, a review J Psychiatr Res 2006 40 6 550 567 10.1016/j.jpsychires.2005.08.002 16214171

[B34] Pariante CM Lightman SL The HPA axis in major depression: classical theories and new developments Trends Neurosci 2008 31 9 464 468 10.1016/j.tins.2008.06.006 18675469

[B35] Kendler KS Kessler RC Walters EE MacLean C Neale MC Heath AC Eaves LJ Stressful life events, genetic liability, and onset of an episode of major depression in women Am J Psychiatry 1995 152 6 833 842 7755111 10.1176/ajp.152.6.833

[B36] Post RM Transduction of psychosocial stress into the neurobiology of recurrent affective disorder Am J Psychiatry 1992 149 8 999 1010 1353322 10.1176/ajp.149.8.999

[B37] Muigg P Hoelzl U Palfrader K Neumann I Wigger A Landgraf R Singewald N Altered brain activation pattern associated with drug-induced attenuation of enhanced depression-like behavior in rats bred for high anxiety Biol Psychiatry 2007 61 6 782 796 10.1016/j.biopsych.2006.08.035 17224133

[B38] Stone EA Lehmann ML Lin Y Quartermain D Depressive behavior in mice due to immune stimulation is accompanied by reduced neural activity in brain regions involved in positively motivated behavior Biol Psychiatry 2006 60 8 803 811 10.1016/j.biopsych.2006.04.020 16814258

[B39] Pezuk P Aydin E Aksoy A Canbeyli R Effects of BNST lesions in female rats on forced swimming and navigational learning Brain Res 2008 1228 199 207 10.1016/j.brainres.2008.06.071 18619949

[B40] Pezuk P Goz D Aksoy A Canbeyli R BNST lesions aggravate behavioral despair but do not impair navigational learning in rats Brain Res Bull 2006 69 4 416 421 10.1016/j.brainresbull.2006.02.008 16624673

[B41] Schulz D Canbeyli RS Lesion of the bed nucleus of the stria terminalis enhances learned despair Brain Res Bull 2000 52 2 83 87 10.1016/S0361-9230(00)00235-5 10808077

[B42] Rangel A Gonzalez LE Villarroel V Hernandez L Anxiolysis followed by anxiogenesis relates to coping and corticosterone after medial prefrontal cortical damage in rats Brain Res 2003 992 1 96 103 10.1016/j.brainres.2003.08.038 14604777

[B43] Jalabert M Aston-Jones G Herzog E Manzoni O Georges F Role of the bed nucleus of the stria terminalis in the control of ventral tegmental area dopamine neurons Prog Neuropsychopharmacol Biol Psychiatry 2009 33 8 1336 1346 10.1016/j.pnpbp.2009.07.010 19616054 PMC3635540

[B44] Kretz R Local cobalt injection: a method to discriminate presynaptic axonal from postsynaptic neuronal activity J Neurosci Methods 1984 11 2 129 135 10.1016/0165-0270(84)90030-X 6090819

[B45] Cryan JF Markou A Lucki I Assessing antidepressant activity in rodents: recent developments and future needs Trends Pharmacol Sci 2002 23 5 238 245 10.1016/S0165-6147(02)02017-5 12008002

[B46] Cryan JF Valentino RJ Lucki I Assessing substrates underlying the behavioral effects of antidepressants using the modified rat forced swimming test Neurosci Biobehav Rev 2005 29 4-5 547 569 10.1016/j.neubiorev.2005.03.008 15893822

[B47] Porsolt RD Anton G Blavet N Jalfre M Behavioural despair in rats: a new model sensitive to antidepressant treatments Eur J Pharmacol 1978 47 4 379 391 10.1016/0014-2999(78)90118-8 204499

[B48] Porsolt RD Le Pichon M Jalfre M Depression: a new animal model sensitive to antidepressant treatments Nature 1977 266 5604 730 732 10.1038/266730a0 559941

[B49] Joca SR Guimaraes FS Inhibition of neuronal nitric oxide synthase in the rat hippocampus induces antidepressant-like effects Psychopharmacology (Berl) 2006 185 3 298 305 10.1007/s00213-006-0326-2 16518647

[B50] Paxinos G Watson C The rat brain in stereotaxic coordinates 1997 3 Sidney Australia Academic Press 10.1016/0165-0270(80)90021-7 6110810

[B51] Crestani CC Alves FH Tavares RF Correa FM Role of the bed nucleus of the stria terminalis in the cardiovascular responses to acute restraint stress in rats Stress 2009 12 3 268 278 10.1080/10253890802331477 18850495

[B52] Dumont EC What is the bed nucleus of the stria terminalis? Prog Neuropsychopharmacol Biol Psychiatry 2009 33 8 1289 1290 10.1016/j.pnpbp.2009.07.006 19602427 PMC4011829

[B53] Alves FH Crestani CC Resstel LB Correa FM Bed nucleus of the stria terminalis N-methyl-D-aspartate receptors and nitric oxide modulate the baroreflex cardiac component in unanesthetized rats J Neurosci Res 2009 87 7 1703 1711 10.1002/jnr.21974 19156861

[B54] Cecchi M Khoshbouei H Javors M Morilak DA Modulatory effects of norepinephrine in the lateral bed nucleus of the stria terminalis on behavioral and neuroendocrine responses to acute stress Neuroscience 2002 112 1 13 21 10.1016/S0306-4522(02)00062-3 12044468

[B55] Khoshbouei H Cecchi M Morilak DA Modulatory effects of galanin in the lateral bed nucleus of the stria terminalis on behavioral and neuroendocrine responses to acute stress Neuropsychopharmacology 2002 27 1 25 34 10.1016/S0893-133X(01)00424-9 12062904

[B56] Maier SF Role of fear in mediating shuttle escape learning deficit produced by inescapable shock J Exp Psychol Anim Behav Process 1990 16 2 137 149 10.1037/0097-7403.16.2.137 2335769

[B57] Maier SF Grahn RE Kalman BA Sutton LC Wiertelak EP Watkins LR The role of the amygdala and dorsal raphe nucleus in mediating the behavioral consequences of inescapable shock Behav Neurosci 1993 107 2 377 388 10.1037/0735-7044.107.2.377 8484901

[B58] Morilak DA Cecchi M Khoshbouei H Interactions of norepinephrine and galanin in the central amygdala and lateral bed nucleus of the stria terminalis modulate the behavioral response to acute stress Life Sci 2003 73 6 715 726 10.1016/S0024-3205(03)00392-8 12801593

[B59] Sherman AD Sacquitne JL Petty F Specificity of the learned helplessness model of depression Pharmacol Biochem Behav 1982 16 3 449 454 10.1016/0091-3057(82)90451-8 7200610

[B60] Graeff FG Guimaraes FS De Andrade TG Deakin JF Role of 5-HT in stress, anxiety, and depression Pharmacol Biochem Behav 1996 54 1 129 141 10.1016/0091-3057(95)02135-3 8728550

[B61] Sapolsky RM Stress and plasticity in the limbic system Neurochem Res 2003 28 11 1735 1742 10.1023/A:1026021307833 14584827

[B62] Jefferys D Funder J Nitric oxide modulates retention of immobility in the forced swimming test in rats Eur J Pharmacol 1996 295 2-3 131 135 10.1016/0014-2999(95)00655-9 8720576

[B63] Jefferys D Funder JW The forced swimming test: effects of glucose administration on the response to food deprivation and adrenalectomy Eur J Pharmacol 1991 205 3 267 269 10.1016/0014-2999(91)90908-9 1817963

[B64] Veldhuis HD De Korte CC De Kloet ER Glucocorticoids facilitate the retention of acquired immobility during forced swimming Eur J Pharmacol 1985 115 2-3 211 217 10.1016/0014-2999(85)90693-4 4065208

